# *Lentinula edodes* Sing Polysaccharide: Extraction, Characterization, Bioactivities, and Emulsifying Applications

**DOI:** 10.3390/foods12173289

**Published:** 2023-09-01

**Authors:** Yan Dai, Lei Wang, Xingyi Chen, Angxin Song, Laping He, Lingyuan Wang, Diandian Huang

**Affiliations:** 1 College of Liquor and Food Engineering, Guizhou University, Guiyang 550025, Chinaxychen3@gzu.edu.cn (X.C.); axsong@gzu.edu.cn (A.S.); helaping@163.com (L.H.);; 2 Key Laboratory of Agricultural and Animal Products Storage & Processing of Guizhou Province, Guizhou University, Guiyang 550025, China

**Keywords:** *Lentinula edodes* Sing, polysaccharide, emulsifying properties, antioxidant properties, hypoglycemic activity

## Abstract

In the present work, the optimization of extraction, emulsifying properties, and biological activities of polysaccharides from *Lentinula edodes* Sing (LES) were studied. The results showed LES polysaccharides extracted by hot water or ultrasonication are a group of β-glucan. Among all the samples, the one extracted by hot water showed the best emulsifying capacity. In addition, the results demonstrated that LES polysaccharide had strong scavenging activities in vitro on DPPH and ABTS radicals, which reached the highest level for the one extracted by 90 min ultrasonication (*p* < 0.05). Overall, *Lentinula edodes* Sing polysaccharides (LESPs) may have potential applications as emulsifying agents in food industries.

## 1. Introduction

*Lentinula*, a genus of the family Marasmiaceae, which is best known for the edible and medicinal *Lentinula edodes* originating from East Asia, is widely cultivated in Asia, Europe, Australia, and North and South America [1,2]. *Lentinula edodes* is now the second most popular edible fungi in the world owing to its desirable taste and multiple functional activities [3]. One of the major ingredients which contribute to *Lentinula edodes*’ bioactive function is the polysaccharide fraction, which has shown antioxidant, antibacterial, immunomodulatory, antitumor, and antiinflammation effects, etc. [4]. For example, M et al. extracted and isolated linear (1→6)-β-d-glucan G-1 from *Lentinula edodes*. The IC50 of 2.2-diphenyl;-1 picrylhydrazine (DPPH) is 183.8 μg/mL and its antioxidant activity is higher than other fungal polysaccharides [5]. Moreover, Murphy et al. found through an in vitro lung injury model that Lentinan effectively attenuated the production of proinflammatory cytokines (IL-2, IL-6, IL-8, and TNF-α), while significantly increasing the secretion of anti-inflammatory cytokine IL-10 [6]. HPLPS, another polysaccharide isolated and purified from the fruiting bodies of *Lentinula edodes*, and reduced the proliferation of HepG2 and HeLa cells in a dose-dependent manner [7]. However, as a subfamily member of *Lentinula*, *Lentinula edodes* Sing (LES, referred to as “Masang fungus” in Chinese), is a kind of wild mushroom unique to Southwest China that grows on the dead branches of the *Coriaria nepalensis* Wall in spring and autumn [8,9]. LES has only been domesticated and planted in recent years, so there are few related studies, especially of its polysaccharides, which have not been studied [10,11]. The extraction of edible fungi polysaccharide using hot water is a conventional and simple method but it possesses some disadvantages like long extraction time, high energy requirement, and less extraction yield [12,13]. Ultrasound-assisted extraction (UAE) is currently considered an efficient and environmentally friendly method for extracting polysaccharides from various plant sources [14,15]. Ultrasonic waves can disrupt the edible fungi cell walls by the cavitation effect, resulting in the mass transfer between the solvent and the material to extract [16]. However, polysaccharides in natural products are extracted with hot water and UAE, which have different molecular weights, chemical compositions, and molecular conformations, such as the presence and location of functional groups and linkage patterns [17,18]. There are parameters that significantly influence polysaccharide extraction efficiency and biological activity through different ultrasonic conditions, such as ultrasonic power [19]. The result is a difference in the physiological activities of polysaccharides derived from different extraction methods [20]. Currently, UAE has widely used the extraction process of polysaccharides from various natural products (such as mushrooms) to improve the yield of target compounds [16].

Alzorqi et al. reported on the polysaccharides obtained by UAE from the *Ganoderma lucidum* that have a higher molecular weight and a better branching degree, as well as better in vitro antioxidant activity compared to traditional hot water extraction [21]. The lentinan normally has a high molecular weight, ranging from 1.16 × 10^3^ to 6.84 × 10^3^ kDa, which includes glucose, galactose, mannose, and arabinose with different molar ratios [1]. Additionally, the polysaccharides that contained β-glycoside bonds or with low molecular weight showed higher antioxidant bioactivity than other fungal polysaccharides [5,22]. Until now, there is little information on the physiochemical characteristics and the functional activities of the LES polysaccharide. The polysaccharides were extracted through hot water extraction and UAE, after which four polysaccharides were obtained, namely H90, U10, U20, and U60. Through the use of chemical and structural characteristics, antioxidant activity, hypoglycemic activity, and antilipid digestion studies, it is expected to provide a theoretical basis for the LES polysaccharide.

## 2. Materials and Methods

### 2.1. Materials and Reagents

The dried *Lentinula edodes* Sing was obtained from Guangzhou Extraction Biotechnology Co., Ltd., Guangzhou, China. A 30-g sample was pulverized with a miniature plant grinder (FSJ-A05N6, Guangdong Bear Electric Co., Ltd., Guangzhou, China) at low speed for one minute and the next pulverization was continued after the temperature was cooled to room temperature, and so repeated several times. The powder is passed through a 100-mesh sieve (aperture about 150 μm), and the part larger than the pore size in the sample is recrushed. Finally, the sample powder with a particle size of less than 150 μm was vacuum-packed and stored at room temperature until analysis and extraction. The sample powder was then vacuum packed and stored at room temperature until analysis and extraction.

Phenol, KBr (spectroscopic-grade), D-galacturonic acid, D-glucose, ABTS [2,2′-Azino-bis (3-ethylbenzothiazoline-6-sulfonic acid) 2,2-diphenyl-1-picrylhydrazyl (DPPH), and monosaccharide standards were purchased from Sigma-Aldrich (Shanghai) Trading Co., Ltd. (Shanghai, China). The acarbose, α-amylase (from the porcine pancreas), α-Glucosidase (from yeast), and 4-nitrophenyl-α-D-glucopyr-anoside (pNPG) were obtained from Shanghai Yuanye Bio-Technology Co., Ltd. (Shanghai, China). Other chemical reagents used in this study were of analytical grade, most of which were purchased from Sinopharm Chemical Reagent Co., Ltd. (Shanghai, China).

### 2.2. Ultrasound-Assisted Extraction

In this study, the ultrasound-assisted extraction of polysaccharides from LES was based on the method of Mao et al. with appropriate modifications [23]. Briefly, the pretreated sample powder was mixed with distilled water at the liquid–solid ratio of 30:1 and then extracted in a SCIENTZ-IID processor with a maximum output power of 600 W (Ningbo Scientz Biotechnology Co., Ltd., Ningbo, China). The ultrasound treatment of LES was performed at a fixed processor power of 80% amplitude (480 W) for a selected effective ultrasonic time of 10, 20, and 60 min. During the ultrasound treatment, one hundred milliliters of the LES solution was contained in a 250 mL glass beaker, which was immersed in ice water to keep the solution temperature below 50 °C.

### 2.3. Hot Water Extraction

As the comparison group, hot water extraction of the polysaccharides from LES followed the method previously reported by Akram et al., with slight modifications [24]. Briefly, ten grams of pretreated sample were mixed with 300 mL of water and then extraction with hot water was carried out directly for 1.5 h in a water bath at 90 °C (repeated two times) without being pretreated with the ultrasound processor.

The insoluble material was separated from the solution by centrifugation for 15 min at 8000 rpm. The supernatant was concentrated to one quarter of its original volume and precipitated overnight at 4 °C with four volumes of ethanol. After another centrifugation (8000 rpm for 15 min), the precipitate was dissolved by the addition of a tenfold volume of water and lyophilized (Model FD-1C-50, Shanghai Shun Zhi Instrument Manufacturing Co., Ltd., Shanghai, China) to obtain polysaccharides. The samples were named H90, U10, U20, and U60 according to the ultrasonic time from small to large. The extraction yield (%) of polysaccharides was calculated according to Equation (1):

(1) $$\mathrm{Yield}\;\left(\%\right)=\frac{\mathrm{weight}\;\mathrm{of}\;\mathrm{the}\;\mathrm{dried}\;\mathrm{crude}\;\mathrm{polysaccharide}\;\left(g\right)}{\mathrm{weight}\;\mathrm{of}\;\mathrm{powder}\;\left(g\right)}\times 100\%$$

### 2.4. Chemical Composition Analysis

The contents of neutral sugar, uronic acid, total phenolic, and protein were determined according to methods including the phenol–sulfuric acid method [25], 3-hydroxy diphenyl [26], Folin–Ciocalteu [27], and bicinchoninic acid [28].

The monosaccharide composition of LESPs was determined by ion chromatography (ICS 5000^+^, Thermo Fisher Scientific, Waltham, MA, USA) equipped with Dionex™ CarboPac™ PA20 (150 × 3.0 mm, 10 μm) liquid chromatography column and the injection volume was 5 μL. The appropriate amount of sample was hydrolyzed with 1 mL trifluoroacetic acid (TFA, 2 M) at 121 °C for 2 h and blown dry with nitrogen. Add 99.99% methanol to the wash, then blow dry, and repeat the methanol wash 2 to 3 times. Add sterile water to dissolve and transfer to the chromatographic bottle for testing. For mobile phase A (H_2_O), mobile phase B (0.1 M NaOH), and mobile phase C (0.1 M NaOH, 0.2 M NaAC), the column temperature was 30 °C and the monosaccharide components were analyzed and detected by an electrochemical detector.

### 2.5. Determination of Color Differences

A colorimeter (NH350, Zhejiang Top Cloud-Agri Technology Co., Ltd., Hangzhou, China) was used to measure the color of different *Lentinula edodes* Sing polysaccharide samples [29,30]. The instrument was calibrated using black and white tiles. The L* (lightness), a* (redness), and b* (yellowness) values were measured three times. The calculation method of the whiteness index (WI) and total color difference (ΔE*) of each sample is as follows:

(2) $$WI=100-\sqrt{{\left(100-L^{*}\right)}^{2}+{a^{*}}^{2}+{b^{*}}^{2}}$$

(3) $$\Delta E^{*}=\sqrt{{\left(L^{*}-L_{0}^{*}\right)}^{2}+{\left(a^{*}-a_{0}^{*}\right)}^{2}+{\left(b^{*}-b_{0}^{*}\right)}^{2}}$$

where $L_{0}^{*}$, $a_{0}^{*}$, and $b_{0}^{*}$ were the chromaticity values of the blank sample.

### 2.6. Fourier Transform Infrared (FTIR) Spectroscopy

The FTIR spectra of LES polysaccharides were analyzed by FT-IR spectroscopy (PerkinElmer Frontier, Waltham, MA, USA). The polysaccharide sample was mixed with the dried spectroscopic-grade KBr powder (baked at 400 °C for 30 min, the ratio of the KBr/sample is 100/1) and then pressed into a tablet for FT-IR spectroscopy measurement. The scanning range is 4000 to 400 cm^−1^, the resolution is 4 cm^−1^, and the number of scans is 32.

### 2.7. Scanning Electron Microscope (SEM)

Dried samples prepared by scanning thin gold films were scanned using a scanning electron microscope (TM3030, HITACHI, Tokyo, Japan) with an accelerating voltage of 15.0 kV; then, the polysaccharide samples were photographed and observed with 800× and 3000× magnifications.

### 2.8. Emulsifying Properties

The determination of emulsifying properties followed the previous method carried out by Xu et al., with appropriate modification [31]. Briefly, ten milliliters of samples (10 mg/mL, soaked overnight) were mixed with 10 mL of sunflower oil in a 50 mL centrifuge tube, using a high-speed shear homogenizer at 10,000 rpm, for 2 min. The mixture was placed at room temperature for 30 min. The emulsion volume was measured and the microscopic morphology was observed. The emulsion capacity was calculated according to Equation (4):

(4) $$\mathrm{The}\;\mathrm{emulsion}\;\mathrm{capacity}\;(\%)=\frac{\mathrm{Height}\;\mathrm{of}\;\mathrm{emulsion}\;\mathrm{layer}}{\mathrm{total}\;\mathrm{height}\;\mathrm{of}\;\mathrm{fluid}}\times 100$$

### 2.9. Congo Red Test and Iodine-Potassium Iodide Analysis

The helical structure of polysaccharides was determined by the Congo red test, which was based on the method of Mutaillifu et al. with appropriate modifications [32]. Briefly, one milliliter of the Congo red solution (100 μmol/L) was mixed with 1.0 mL of polysaccharide sample solution (1.0 mg/mL); then, water and NaOH solution (1.0 mol/L) were added to the total volume of 4 mL by adjusting the NaOH final concentration of 0, 0.05, 0.10, 0.15, 0.20, 0.25, 0.30, 0.35, and 0.40 mol/L, respectively. As a control, Congo red solution was mixed with the same concentration of NaOH solution. After incubating at room temperature for 5 min, the solutions were scanned at the wavelength of 400–600 nm using a microplate reader (SuperMax-3100, Shanghai Flash Spectrum Biological Technology Co., Ltd., Shanghai, China). Iodine-potassium iodide analysis was performed by reacting polysaccharides with 0.2% potassium iodide solution (containing 0.02% iodine) and measuring the sweeping spectrum at 300–700 nm [33].

### 2.10. Antioxidant Activity

The antioxidant activity of the sample was measured using a DPPH, ABTS^+^, and TPTZ assay. The tested samples were prepared at different concentrations (0.05, 0.1, 0.2, 0.4, 0.8, and 1.6 mg/mL). VC was used as the positive control.

#### 2.10.1. Scavenging Capacity of DPPH Free Radicals

The determination of DPPH free radical scavenging activity followed the previous method carried out by Zhou et al., with appropriate modification [34]. Briefly, three hundred microliters of DPPH· methanol solution (0.1 mM) were mixed with 100 μL of samples and incubated at room temperature for 30 min in the dark. Then, the absorbance was measured at 517 nm using a microplate reader (SuperMax-3100, Shanghai Flash Spectrum Biological Technology Co., Ltd., Shanghai, China). The DPPH· scavenging activity was calculated according to Equation (2):

(5) $$\mathrm{The}\;\mathrm{DPPH}\cdot \;\mathrm{scavenging}\;\mathrm{rate}\;(\%)=\left(1-\frac{{A_{2}-A}_{1}}{A_{0}}\right)\;\times \;100$$

where A_2_ was the absorbance of the mixtures, with A_1_ without DPPH·, and A_0_ without sample. The absence was substituted by an equivalent solvent.

#### 2.10.2. Scavenging Capacity of ABTS Free Radicals

The determination of ABTS free radical scavenging activity followed the previous method carried out by Wang et al., with appropriate modification [35]. Briefly, the ABTS^+^ solution (7.0 mM) was mixed with an equal volume of K_2_S_2_O_8_ solution (2.45 mM). The ABTS^+^ working solution was configured following incubation at 4 °C for 16 h in darkness and was diluted with PBS (pH = 7.4) to the absorbance value at 734 nm as 0.70 ± 0.05. Fifty microliters of sample solution were mixed with 150 μL of ABTS^+^ working fluids and incubated at room temperature for 5 min in the dark and the absorbance was measured at 734 nm. The ABTS^+^ scavenging activity was calculated according to Equation (3):

(6) $$\mathrm{The}\;{\mathrm{ABTS}}^{+}\;\mathrm{scavenging}\;\mathrm{rate}\;(\%)=\left(1-\frac{{A_{2}-A}_{1}}{A_{0}}\right)\times 100$$

where A_2_ was the absorbance of mixtures, with A_1_ without ABTS^+^ and A_0_ without sample. The absence was substituted by an equivalent solvent.

#### 2.10.3. Ferric-Reducing Antioxidant Power (FRAP)

The FRAP of polysaccharides was determined by a T-AOC Assay Kit (Macklin Biochemical Co., Ltd., Shanghai, China) [21]. Briefly, the FRAP reagent was freshly prepared by mixing acetate buffer, TPTZ solution, and ferric chloride at the volume ratio of 7:1:1. Then, thirty microliters of sample solution and 90 μL of distilled water were added to 900 μL of the FRAP reagent. The mixture was kept at 37 °C for 10 min in the dark and the absorbance was measured at 593 nm. An FeSO_4_ solution (0.003125–0.1 μM) was used for the standard curve (y = 11.119x − 0.0074, R^2^ = 0.9988) and the FRAP value was calculated according to the Equation (4):

(7) $$\mathrm{FRAP}\;\mathrm{value}\;\mathrm{of}\;\mathrm{polysaccharides}\left(\mu \mathrm{mol}/\mathrm{mg}\right)=34\times \frac{x}{\mathrm{Cpr}}$$

where x is calculated from the standard curve, and Cpr is the sample concentration.

### 2.11. Assay for Hypoglycemic Activity

The hypoglycemic activity of the LESPs was characterized by measuring α-amylase, α-glucosidase inhibitory activity, and the glucose adsorption capacity. Acarbose was used as the positive control.

#### 2.11.1. Inhibition of the α-Amylase Activity

The inhibitory activity of LESPs on α-amylase was determined according to Niu et al., with appropriate modification [36]. Briefly, five hundred microliters of sample solutions of different concentrations (0.1, 0.2, 0.4, 0.8, 1.6, and 3.2 mg/mL) were mixed with 500 µL of α-amylase solution (1 U/mL) and incubated at 25 °C for 10 min. Then, 500 µL of starch solution (1%, *w/v*) were added. The enzyme reaction was terminated by adding 1 mL of DNS reagent after incubation for 10 min at 25 °C, followed by incubation in a boiling water bath for 5 min. The reaction mixture was diluted by adding 10 mL of distilled water. The absorbance of the mixture was measured at 540 nm using a multimode microplate reader. The inhibition rate was calculated using the following formula:

(8) $$\alpha \text{-}\mathrm{Amylase}\;\mathrm{activity}\;\mathrm{inhibition}\;(\%)=(1-\frac{A_{2}-A_{1}}{A_{0}})\;\times \;100$$

where A_2_ was the absorbance of mixtures, with A_1_ without α-Amylase, and A_0_ without sample. The absence was substituted by equivalent PBS.

#### 2.11.2. Inhibition of the α-Glucoside Activity

The inhibitory activity of LESPs on α-amylase was determined according to Niu et al., with appropriate modification [37]. Briefly, fifty microliters of sample solutions of different concentrations (0.1, 0.2, 0.4, 0.8, 1.6, and 3.2 mg/mL) were mixed with 100 µL of 5 mM α-glucosidase solution (0.5 U/mL) and incubated at 37 °C for10 min. Then, 100 µL of 4-nitrophenyl-α-D-glucopyranoside (pNPG) were added. The enzyme reaction was terminated by adding 1 mL of 1 M sodium carbonate solution after incubation for 20 min at 37 °C. Measure the absorbance of the mixture at 405 nm using a multimode microplate reader. The inhibition rate was calculated using the following formula:

(9) $$\alpha \text{-}\mathrm{glucosidase}\;\mathrm{activity}\;\mathrm{inhibition}\;(\%)=(1-\frac{A_{2}-A_{1}}{A_{0}})\;\times \;100$$

where A_2_ was the absorbance of mixtures, with A_1_ without α-glucosidase, and A_0_ without sample. The absence was substituted by equivalent PBS.

#### 2.11.3. Glucose Adsorption Capacity (GAC)

Two-hundred fifty mg of polysaccharide were dissolved in 25 mL of glucose solution (100 mmol/L) and placed in a water bath at 37 °C for 6 h [38]. Then, it was centrifuged at 3000 rpm for 15 min and the glucose content in the supernatant was measured by the DNS method.

In the same way, distilled water was used instead of glucose solution as the sample blank. A glucose solution without a sample was used as the control experiment. Guar gum was used as a positive control. The GAC was calculated using the following formula:

(10) $$\mathrm{GAC}(\mathrm{mg}/g)=\frac{G_{1}-(G_{2}-G_{3})}{W}\times V$$

where G1, G2, and G3 are the glucose concentration in the supernatant of the control experiment, the sample experiment, and the blank group, respectively (mg/mL); W is the weight of the sample (g); V is the volume of the mixture (25 mL).

### 2.12. Effects of Polysaccharides on the Lipolysis In Vitro Digestion Model

#### 2.12.1. Polysaccharide Stock Solutions Preparation

Two-hundred fifty mg of each polysaccharide sample (H90, U10, U20, and U60) were dispersed into 50 mL of distilled water to prepare a stock solution (0.5%, *w/v*). The solution was then stirred overnight at room temperature at 300 rpm to ensure complete dissolution of polysaccharide.

#### 2.12.2. Stock Emulsion Preparation

Two hundred g of 2.5% (*w/v*) tween 80 emulsifier solution were used to emulsify 50 g of corn oil with a high-speed homogenizer at 16,000 rpm for 10 min (homogenization for 2 min at intervals of 3 min and homogenization for 5 times in total) to prepare a crude oil emulsion containing 20% (*w/w*) corn oil.

#### 2.12.3. Polysaccharide–Emulsion Mixture Preparation

The polysaccharide solution was mixed with a crude oil emulsion (9:1, *w/w*) and stirred at 500 rpm for 2 h at room temperature to obtain a polysaccharide–lipid emulsion. The lipid emulsion prepared with distilled water instead of polysaccharide solution was used as a control.

#### 2.12.4. Static In Vitro Digestion Model

In vitro digestion using a simulated electrolyte solution and enzymes according to methods reported in the literature [39,40]. Simulated saliva fluid (*SSF*) was simulated by dissolving 0.375 g NaCl, 0.075 g CaCl_2_, and 0.75 g KCl in 500 mL deionized water and adjusting the pH to 6.80 with a 0.1 M NaOH solution. Simulated gastric fluid (*SGF*), comprising 1.6 g of sodium chloride, 0.075 g of CaCl_2_, 0.55 g of potassium chloride, and 0.3 g of NaHCO_3_, was dissolved in 500 mL of deionized water and, then, the pH was adjusted to 2.5 with 1 M HCl solution. Simulated intestine fluid (*SIF*) was prepared by dissolving 2.7 g of sodium chloride, 0.175 g of CaCl_2_, and 0.3265 g of KCl in 500 mL of deionized water and, then, the pH value was adjusted to 7.0 with 0.1 M NaOH solution.

The digestion model consists of three stages of simulated oral digestion, gastric digestion, and intestinal digestion [41]. First, mix the polysaccharide–lipid emulsion with SSF (1:1, *w/w*), adjust the pH to 6.8 using 0.1 M sodium hydroxide or hydrochloric acid solution, add α-amylase (50 U/mL), and incubate with constant agitation at 100 rpm for 10 min at 37 °C. Mix the saliva phase obtained after incubation with SGF (1:1, *w/w*), adjust the pH to 2.5 using 0.1 M hydrochloric acid solution, add pepsin (400 U/mL), and incubate at 37 °C with continuous stirring at 100 rpm for 2 h. The gastric phase obtained after incubation was then mixed with SIF (1:1, *w/w*), bile salts (10 mM) were added, the pH was adjusted to 7.0 using a 0.1 M NaOH solution, and incubated at 37 °C with constant agitation at 100 rpm for 2 h. Meanwhile, sodium hydroxide solution (0.05 M) was added in a dropwise manner to maintain a constant pH of 7.0, and the volume of sodium hydroxide solution added changed with time; then, the concentration of free fatty acid (FFA) produced by lipolysis was calculated. The release of FFA (%, *w/w*) was calculated using the following formula [41]:

(11) $$F\mathrm{FA}(\%)=\left(\frac{V_{Na\mathrm{OH}}\times C_{\mathrm{NaOH}}\times {\mathrm{MW}}_{\mathrm{Lipid}}}{2\times W_{\mathrm{Lipid}}}\right)\times 100$$

where V_NaOH_ is the volume used to neutralize FFA with sodium hydroxide (0.05 M) during the titration reaction; C_NaOH_ is the concentration of sodium hydroxide solution (0.05 M); MW_Lipid_ is the average molecular weight of corn oil (872 g/mol), and W_Lipid_ is the initial weight of corn oil in the enteric phase.

### 2.13. Statistical Analysis

All experiments were performed in triplicate and the corresponding data were expressed as means ± standard deviation (SD). One-way ANOVA was performed by *t*-test and Tukey’s test using SPSS (Version 16; SPSS Inc., Chicago, IL, USA), where the probability value of *p* < 0.05 was considered to be statistically significant.

## 3. Results

### 3.1. Physicochemical Characterizations Analysis

The content of reducing sugar, polyphenol, protein, uronic acid, and yield of H90, U10, U20, and U60 are given in Table 1.

### 3.2. Monosaccharide Composition Analysis

Table 2 shows the monosaccharide composition of the polysaccharides obtained by four extraction conditions. According to the table, it can be seen that PSP1 contains a rich monosaccharide composition. Among them, glucose, galactose, and mannose are the main monosaccharides, which is consistent with the lentinan extracted by Zhao et al. [2].

### 3.3. Determination of Appearance Difference

Polysaccharides could be incorporated into food systems due to their many excellent properties to produce functional foods. However, the dark color of polysaccharides could be unfavorable to the quality of food, and dark-colored polysaccharides should be avoided. Table 3 shows the color differences of the polysaccharide freeze-dried powder obtained at different ultrasonic treatment times during the polysaccharide extraction process. The ultrasonic treatment times significantly influenced the color (*p* < 0.05). The highest whiteness index is the ultrasonic treatment for 20 min (82.96 ± 0.37), and the darkest color is the ultrasonic treatment for 60 min (79.91 ± 0.25). The difference in the color of extracted polysaccharides might be related to the length of the sonication time of the extraction process. The ultrasonic treatment can contribute to the whiteness index. With the increase of ultrasonic time, the temperature of the center of the system gradually increases, which promotes the progress of chemical reactions such as Maillard reactions. Eventually, the polysaccharide darkens in color. Furthermore, the total color difference (ΔE*) between the standard color plate and polysaccharide samples was calculated; it was observed that the smaller the whiteness index, the larger the value of ΔE*.

### 3.4. Analysis of Fourier Transform Infrared (FTIR) Spectroscopy, Congo Red Analysis, and Iodine-Potassium Iodide

**Figure 1:**
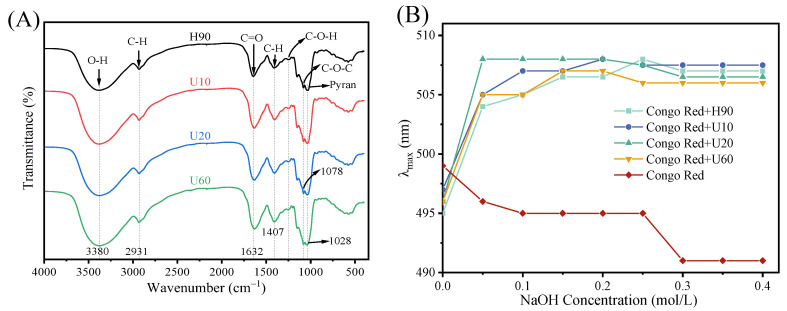
FTIR spectra (**A**) and triple-helical conformation (**B**) analysis of polysaccharides.

As shown in Figure 1A, the FT-IR spectra of H90, U10, U20, and U60 exhibit similar characteristic peaks. All these peaks are characteristics of polysaccharides. Peaks at 3380 and 2931 cm^−1^ are connected to the absorption of the O–H and C–H stretching vibration, respectively [20]. The absorption peak at 1632 cm^−1^ was attributed to the C=O bond, while the peak at 1407 cm^−1^ was attributed to the C–H stretching vibration of the aldehyde group [3,37]. The results were consistent with the results of the monosaccharide composition analysis, which confirmed that the four polysaccharides contained glucuronic acid. In this region of 1028–1246 cm^−1^, weak peaks can be attributed to the stretching vibration of the C–O–C or C–O–H group and indicated that pyran rings could be present in these polysaccharides [42]. The peaks at 847 and 933 cm^−1^ are attributed to α-d-glucans, while the weak absorbance band 889 cm^−1^ symbolizes β-d-glucans [43]. At different ultrasonic times, the FTIR spectra of the four polysaccharides were not significantly different, which may be because the degraded sugar units still existed in the samples.

When the solution contains low NaOH concentrations, the triple-helix conformation of the polysaccharide to which Congo red binds causes a redshift of maximum absorption wavelength (λmax) [44]. The shifts in λmax of the four polysaccharide–Congo red complexes with different concentrations (0–0.40 mol/L) of NaOH were shown in Figure 1B. Compared with the blank solution of Congo red, λmax of the complexes showed an obvious redshift with the change of NaOH concentration, indicating that the polysaccharides have triple-helix conformation (Figure 1A).

**Figure 2:**
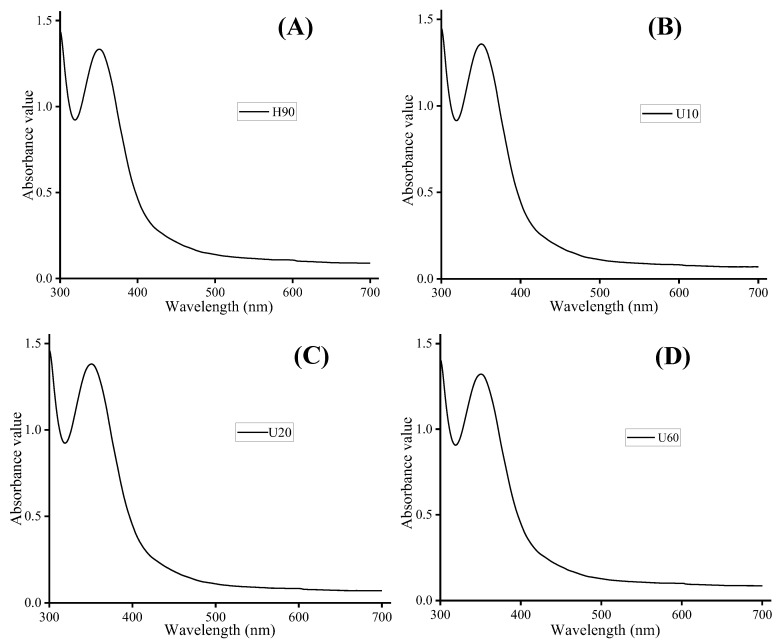
Iodine-potassium iodide analysis of polysaccharides. (**A**–**D**) correspond to samples H90, U10, U20 and U60, respectively.

Figure 2 shows the scanning spectrum of polysaccharide–iodine–potassium iodide complexes. In each of the four polysaccharides, the maximum absorption peak is mainly concentrated near 350 nm, whereas there is no peak at 565 nm, indicating a long side chain and more branches [33].

### 3.5. Emulsifying Properties

**Figure 3:**
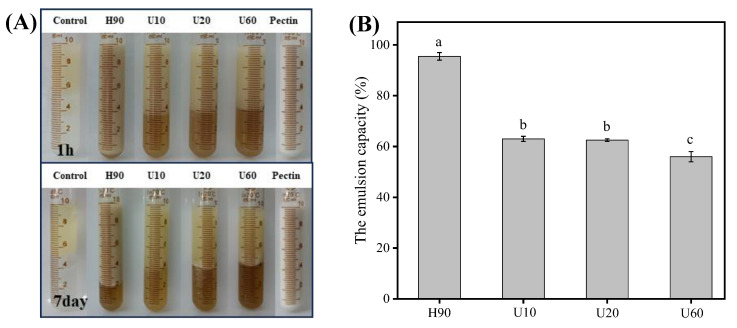
The digital photos (**A**) and emulsion capacity (**B**) of the emulsion. Different letters above columns show significant differences in emulsifying capacity among different samples (*p* < 0.05). Error bars represent standard deviation (*n* = 3).

**Figure 4:**
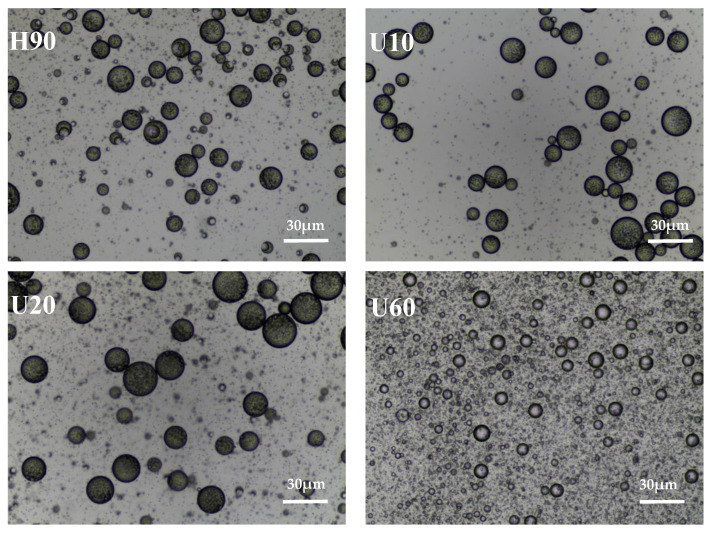
The optical micrographs of emulsions stabilized by LESP.

The digital photos and optical micrographs (Figure 3 and Figure 4) of the prepared emulsion can visually show the emulsifying properties of LESPs. After 1 h of preparation, the H90 extracted by hot water showed slight phase separation, while the polysaccharide emulsified solutions extracted by ultrasonic assistance showed different degrees of phase separation and demulsification. After 7 days, compared with the ultrasonically extracted polysaccharide emulsion, the hot water-extracted polysaccharide solution still maintained a higher emulsion layer height, showing better emulsion stability. Compared with pectin, lentinan has weaker emulsifying activity and stability. It may be because lentinan has a three-dimensional helical structure, has more hydrophilic groups, and lacks surface activity at the oil–water interface [45]. In addition, lentinan is a neutral polysaccharide with less surface charge, which reduces the charge repulsion between droplets, which is not conducive to the formation and stability of emulsions [46].

### 3.6. Scanning Electron Microscope (SEM)

**Figure 5:**
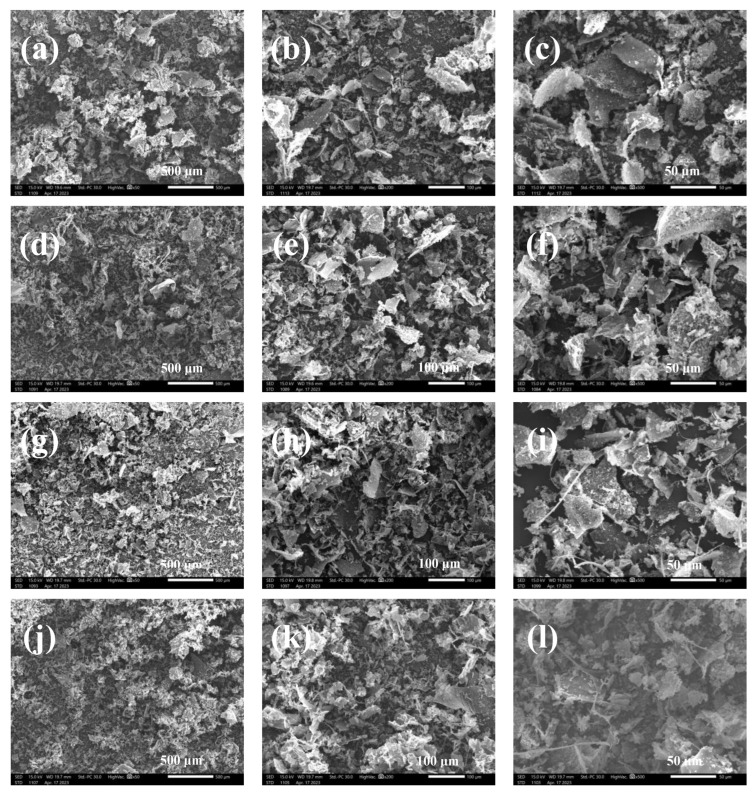
(**a**–**c**) SEM with different magnified images of LESP without ultrasonication treatments and (**d**–**l**) SEM with LESP obtained under different ultrasonication conditions.

Scanning electron microscopy was used to determine the microscopic characteristics of the samples obtained under different extraction conditions. SEM micrographs of LESP samples at different magnifications are presented in Figure 5a–l. The H90 (Figure 5a–c) surface is rough and irregular and its surface is bestrewed dense pores and networks when compared with U10, U20, and U60, indicating that H90 may have a smaller intermolecular force to promote the formation of loose structure [3]. In addition, some bead-like structures were observed from the surfaces of U10, U20, and U60, indicating that U10, U20, and U60 may be used for encapsulation purposes in drug delivery system design [47].

### 3.7. Antioxidant Activity In Vitro

The activities of scavenging DPPH free radicals and ABTS free radicals are two common indicators to reflect the antioxidant capacity [35]. The scavenging activity of LESPs on DPPH free radicals and ABTS free radicals are shown in Figure 5a,b, respectively. All LESPs exhibited concentration-dependent scavenging activity on DPPH free radicals and ABTS free radicals.

**Figure 6:**
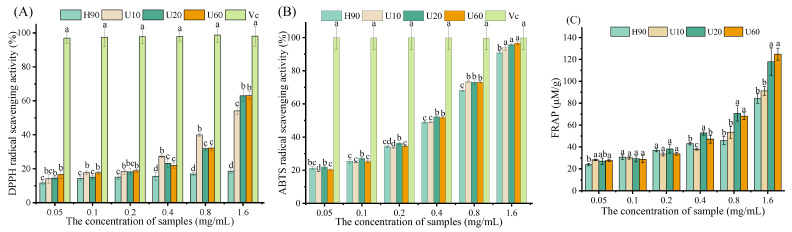
DPPH (**A**), ABTS (**B**), and FRAP (**C**) radical scavenging rate. Different letters above columns show significant differences in antioxidant capacity among different samples (*p* < 0.05). Error bars represent standard deviation (*n* = 3).

When the DPPH free radicals were reduced, the methanol solution of DPPH’s purple color faded to light yellow and the absorbance at 517 nm decreased. The degree of absorbance variation reflects the antioxidant capacity of a substance [18]. As shown in Figure 6A, all LESPs exhibited concentration-dependent scavenging activity on the DPPH radical. But it is not particularly noticeable, and it is very low compared to Vc.

As can be seen from Figure 6B, with the increase of the polysaccharide concentration, the ABTS free radical scavenging rate increased significantly. When the concentration is 1.60 mg/mL, the ABTS free radical scavenging ability is close to that of Vc at the same concentration. Furthermore, the scavenging activity of LESPs on ABTS free radicals showed a descending order of U20 (IC50 = 0.29 mg/mL) > U60 (IC50 = 0.30 mg/mL) > U10 (IC50 = 0.32 mg/mL) > H90 (IC50 = 0.33 mg/mL).

The antioxidant capacity of the iron reduction is expressed in the ferrous sulfate equivalent. As shown in Figure 6C, the iron reduction abilities of LESPs obtained by the four methods are concentration dependent. When the concentration is 1.60 mg/mL, the antioxidant capacity of the iron reduction is 80.27, 91.27, 117.90, and 124.85 μmol/g respectively. It can be seen from the results that, with the increase of ultrasonic time, the iron reduction and antioxidation capacity also gradually increased.

### 3.8. Inhibitory Effects of LES Polysaccharides on α-Amylase and α-Glucosidase Activities

**Figure 7:**
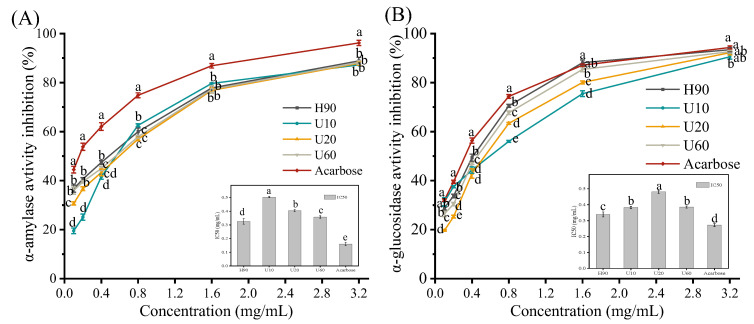
The inhibition effects of LESPs on α-amylase ability (**A**) and α-glucosidase ability (**B**). Different letters above columns show significant differences in enzyme inhibitory activities among different samples (*p* < 0.05). Error bars represent standard deviation (*n* = 3).

α-amylase is widespread among living organisms and α-amylase can hydrolyze starch into oligosaccharides such as maltose. The byproducts of α-amylase hydrolysis are ultimately broken down by other enzymes (such as α-glucosidase) into molecules of glucose, which are rapidly absorbed through the intestinal wall [48]. Inhibition of α-glucosidase, α-amylase, and other carbohydrate-hydrolyzing enzyme activities could reduce the hydrolysis of postprandial carbohydrates, thereby reducing blood glucose levels [49,50]. As shown in Figure 7, the inhibitory effects of LESPs and acarbose on α-amylase and α-glucosidase were concentration-dependent. As documented, the inhibitory effects of natural polysaccharides on α-glucosidase and α-amylase were positively correlated with the contents of some monosaccharides, such as arabinose, xylose, mannose, and uronic acid [51,52,53]. In addition, the hydroxyl and ketone groups of polysaccharides can promote changes in the polarity and molecular conformation of the enzyme, resulting in a partial loss of enzyme activity [36,54]. It can be seen from the Figure that LESPs exerted a better in vitro effect on hypoglycemia. Furthermore, the inhibitory effect of polysaccharide on α-amylase showed a descending order of acarbose (IC_50_ = 0.16 mg/mL) > H90 (IC_50_ = 0.32 mg/mL) > U60 (IC_50_ = 0.36 mg/mL) > U20 (IC_50_ = 0.40 mg/mL) > U10 (IC_50_ = 0.50 mg/mL) (Figure 7A). Furthermore, the inhibitory effect of polysaccharide on α-glucosidase showed a descending order of acarbose (IC_50_ = 0.27 mg/mL) > H90 (IC_50_ = 0.34 mg/mL) > U10 (IC_50_ = 0.38 mg/mL) > U60 (IC_50_ = 0.39 mg/mL) > U20 (IC_50_ = 0.48 mg/mL) (Figure 7B). As indicated by IC50, the inhibitory activity of H90 on α-amylase and α-glucosidase was stronger than U10, U20, and U60. This may be mainly related to the higher content of arabinose, xylose, and mannose in H90. In addition, according to the results of the positive control and the IC50, it was shown that the inhibitory activity of LESPs on α-glucosidase was stronger than α-amylase. It can be seen that polysaccharides have different mechanisms of inhibitory effects on α-glucosidase and α-amylase [36], which may be related to the composition and content of monosaccharides such as arabinose [49,51].

### 3.9. Effects of LES Polysaccharides on Glucose Adsorption Capacity

**Figure 8:**
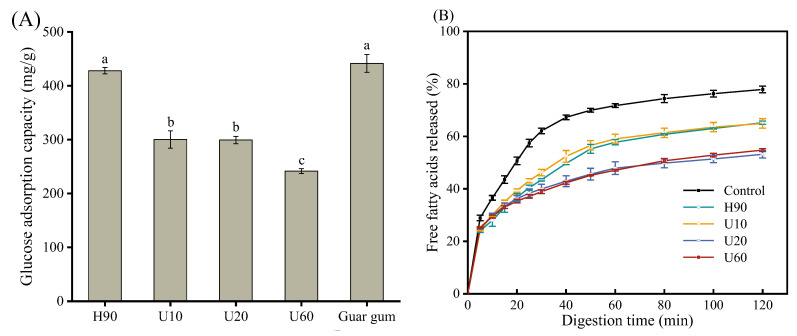
Influence of polysaccharides obtained under different extraction conditions on glucose adsorption capacity (**A**) and in vitro digestion of lipid droplets (**B**). Different letters above columns show significant differences in glucose adsorption capacity among different samples (*p* < 0.05). Error bars represent standard deviation (*n* = 3).

Samples absorb glucose and reduce the digestion and absorption of glucose by the human intestine. It is an effective way to reduce postprandial blood sugar and is widely used to evaluate the hypoglycemic effect of polysaccharides [36]. The glucose adsorption capacity of four polysaccharide samples at the same glucose concentration (100 mM) was shown in Figure 8A, with the guar gum as a positive control. It can be seen from the Figure that all samples can effectively adsorb and bind glucose. Among them, H90 has the strongest glucose adsorption capacity and there is no significant difference with the positive control (guar gum). The GAC of the polysaccharides may be related to the physical structure, such as the internal porosity and surface area of polysaccharides [55]. With the increase of ultrasonic time, the adsorption capacity of polysaccharides to glucose decreased significantly. Combined with the SEM analysis, this may be because the ultrasonic treatment reduces the internal porosity of polysaccharides, thereby reducing the total surface area and the adsorption capacity to glucose. Therefore, we concluded that LESPs could adsorb glucose and reduce its utilization for intestinal absorption, thereby suppressing postprandial hyperglycemia.

### 3.10. Effects of LES Polysaccharides on Lipolysis

Reducing the speed rate and extent of lipid digestion within the small intestine is an effective strategy for the treatment of postprandial spikes in blood lipid levels that occur after the intake of high-fat foods [41]. We, therefore, examined the effect of LESP on lipid digestion. As revealed by the iodine-potassium iodide experiment, our polysaccharide samples do not contain starch. In addition, since the lipase activity in the gastric lumen is significantly lower than that in the duodenal tract, assays in the oral and gastric phases can be omitted [41,56]. The relationship between the release degree of free fatty acids in the reaction system and the digestion time was detected by the titration method during the incubation in the intestinal stage, to judge the influence of polysaccharides on the rate and degree of lipid digestion. The lipolysis of emulsions in the presence and absence of LESPs is shown in Figure 8B. Overall, FFA release increased rapidly at the onset of lipolysis and, then, gradually increased over time. At 120 min, the final degree of lipid digestion was as follows: 77.87 ± 1.27%, 65.21 ± 0.63%, 64.94 ± 1.79%, 53.17 ± 1.43%, and 54.75 ± 0.56% for the control, H90, U10, U20, and U60 samples, respectively. Lipid digestibility and extent were significantly reduced in the presence of lentinan compared to the control group (*p* < 0.05).

## 4. Conclusions

A novel source of polysaccharide from *Lentinula edodes* Sing (LES) was obtained using hot water extraction and ultrasonication for different intervals. According to the results of Congo red, iodine-potassium iodide, and infrared spectrum, all four polysaccharides have rich branched chain structures and three-dimensional helical structures with similar FT-IR spectra. Moreover, it revealed that the extraction condition could significantly (*p* < 0.05) affect the extraction yield and emulsifying properties, as well as other physiochemical and biological properties of LES polysaccharide. To be specific, the polysaccharides obtained by hot water extraction showed a stronger emulsifying ability and stability, as well as stronger hypoglycemic activity in vitro. On the other hand, ultrasonication exhibited a positive impact on the antioxidant capacity of LES polysaccharides when the interval was extended from 10 to 60 min. In addition, ultrasonication increased the resistance of polysaccharides to lipid digestion. The present study showed that *Lentinula edodes* Sing can be a great potential source of polysaccharides, especially the one extracted by hot water for hydrocolloids.

## Figures and Tables

**Table 1:** The yield of *Lentinula edodes Sing* polysaccharides and its chemical composition between four extraction methods.

Sample	Reducing Sugar (%)	Polyphenol (%)	Protein (%)	Uronic Acid (%)	Yield (%)
H90	46.08 ± 0.22 ^a^	1.08 ± 0.01 ^b^	5.32 ± 0.18	4.31 ± 0.09	6.24 ± 0.10 ^d^
U10	45.08 ± 0.28 ^ab^	1.11 ± 0.04 ^b^	5.35 ± 0.25	4.71 ± 0.08	7.83 ± 0.17 ^c^
U20	44.26 ± 0.15 ^b^	1.17 ± 0.02 ^b^	5.76 ± 0.17	4.78 ± 0.10	8.48 ± 0.15 ^b^
U60	45.13 ± 0.19 ^ab^	1.38 ± 0.05 ^a^	6.22 ± 0.61	5.09 ± 0.26	9.43 ± 0.19 ^a^

Note: Data are expressed as the means ± standard deviation of at least three independent replicates. Means followed by different small letters for the same column are significantly different (*p* < 0.05).

**Table 2:** The monosaccharide composition (molar ratio, %) of *Lentinula edodes Sing* polysaccharides.

Sample	Fucose	Rhamnose	Arabinose	Galactose	Glucose	Xylose	Mannose	Glucuronic Acid
H90	2.11%	0.28%	0.19%	8.58%	80.06%	1.04%	6.92%	0.84%
U10	2.26%	0.14%	0.10%	8.94%	80.38%	0.51%	6.84%	0.82%
U20	2.33%	0.14%	0.09%	9.07%	80.15%	0.51%	6.87%	0.85%
U60	2.32%	0.19%	0.10%	9.14%	79.79%	0.44%	6.51%	1.50%

**Table 3:** Effect of ultrasonic treatment on the color values of *Lentinula edodes Sing* polysaccharides.

Sample	*L* *	*a* *	*b* *	WI	ΔE*
H90	83.78 ± 0.14 ^b^	3.58 ± 0.17 ^c^	5.50 ± 0.34 ^c^	82.50 ± 0.21 ^b^	13.08 ± 0.21 ^b^
U10	86.02 ± 0.18 ^a^	4.18 ± 0.06 ^b^	8.94 ± 0.12 ^b^	82.88 ± 0.17 ^ab^	12.83 ± 0.17 ^b^
U20	85.88 ± 0.24 ^a^	4.06 ± 0.14 ^b^	8.62 ± 0.30 ^b^	82.96 ± 0.37 ^a^	12.72 ± 0.38 ^b^
U60	83.32 ± 0.26 ^c^	4.84 ± 0.04 ^a^	10.11 ± 0.12 ^a^	79.91 ± 0.25 ^c^	15.77 ± 0.25 ^a^

Data are expressed as the means ± standard deviation of at least three independent replicates. Means followed by different small letters for the same column are significantly different (*p* < 0.05).

## Data Availability

The data used to support the findings of this study can be made available by the corresponding author upon request.
